# Determination of Time Domain Reflectometry Surface Sensors Sensitivity Depending on Geometry and Material Moisture

**DOI:** 10.3390/s22030735

**Published:** 2022-01-19

**Authors:** Zbigniew Suchorab, Agnieszka Malec, Henryk Sobczuk, Grzegorz Łagód, Izolda Gorgol, Ewa Łazuka, Przemysław Brzyski, Anton Trník

**Affiliations:** 1 Faculty of Environmental Engineering, Lublin University of Technology, 40B Nadbystrzycka Str., 20-618 Lublin, Poland; aga_malec@interia.pl (A.M.); h.sobczuk@pollub.pl (H.S.); g.lagod@pollub.pl (G.Ł.); 2 Faculty of Fundamentals of Technology, Lublin University of Technology, 38 Nadbystrzycka Str., 20-618 Lublin, Poland; i.gorgol@pollub.pl (I.G.); e.lazuka@pollub.pl (E.Ł.); 3 Faculty of Civil Engineering and Architecture, Lublin University of Technology, 40 Nadbystrzycka Str., 20-618 Lublin, Poland; p.brzyski@pollub.pl; 4 Department of Physics, Faculty of Natural Sciences and Informatics, Constantine the Philosopher University in Nitra, Tr. A. Hlinku 1, 94974 Nitra, Slovakia; atrnik@ukf.sk; 5 Department of Materials Engineering and Chemistry, Faculty of Civil Engineering, Czech Technical University in Prague, Thákurova 7, 16629 Prague, Czech Republic

**Keywords:** time domain reflectometry, material moisture, apparent permittivity, non-invasive sensor, sensor sensitivity

## Abstract

The article concerns the electric techniques of moisture detection that are based on the evaluation of the apparent permittivity of the tested medium. The main goal of the research was to evaluate the non-invasive Time Domain Reflectometry (TDR) sensors’ sensitivity by measuring the span of elements and material moisture. To that aim, two non-invasive sensor designs were investigated for their sensitivity in the evaluation of the apparent permittivity value of aerated concrete. Sensors A and B were characterized by the spacing between the measuring elements equal to 30 mm and 70 mm, respectively. The tested samples differed in moisture, ranging between 0 and 0.3 cm^3^/cm^3^ volumetric water content. Within the research, it was stated that in the case of the narrower sensor (A), the range of the sensor equals about 30 mm, and in the case of the wider design (B), it equals about 50 mm. Additionally, it was stated that material moisture influences the range of sensor influence. In the case of the dry and low-saturated material, it was not possible to evaluate the range of sensor sensitivity using the adopted method, whereas the range of sensor signal influence was visible for the moist material.

## 1. Introduction

Time Domain Reflectometry (TDR) uses the measurement of the propagation time of a short electromagnetic pulse within a material sample to determine the apparent permittivity of porous materials, which is highly dependent on water content [1,2,3,4]. By knowing the apparent permittivity, the water content can be calculated with the application of calibration characteristics [5,6,7]. The relative dielectric permittivity (*ε*) of water is about 80.0 and is much higher than for other materials typically applied in the building industry [8,9,10]. The value of relative dielectric permittivity is frequency-dependent and decreases significantly for the frequency above 3 GHz [11,12,13]. The TDR measuring devices often utilize pulse signals with the rise time of approximately 0.3 ns, which corresponds to high-energy Fourier components emitted at around 3 GHz [11]. The pulse rise time influences the TDR probes construction details, which were discussed further. In typical probe constructions, the pulse propagates in a waveguide consisting of conductive rods or wires embedded in the material to be measured [14,15,16]. The design of the TDR probe determines the shape of the reflected signal. The accuracy of the propagation time measurement depends on the probe construction details [7,17,18]. Usually, a probe is connected to a measuring device with a coaxial, high-frequency cable that forms a waveguide for the TDR input pulse [17,18]. The same cable conducts the reflected pulse, traveling back to the measuring device, allowing collection and interpretation of the reflected signal. The probe itself forms a waveguide extending into the material under test, so the electromagnetic field propagates within the material with the velocity dependent on material apparent permittivity [5,6]. There are many TDR probe designs that have been constructed and tested for specific measurement conditions, the required accuracy, and the measured material properties [3,14,19,20,21,22].

The TDR probes consisting of rods for temporary placement or permanent installation in the material to be measured were discussed in [23]. Rods protrude from an insulating holder, which ensures the stability of the construction. On the other side of the holder, a coaxial cable is connected to the rods and provides the signal connection to the measuring apparatus. The construction material of the probe rods, their shape, diameter, length, and finally, the spacing between the rods, influence the performance of the probe for a particular purpose [24]. The rods are constructed of conductive material, usually stainless steel, which is not the best conductor but it enables the probes to operate for a long time in wet, saline porous materials without significant corrosion. The diameter of the rods, according to literature data, varies from 1.3 to 12.7 mm [25]. The length of measuring rods of typical TDR probes varies from 21 to 1000 mm [16] and is limited by the strength of the reflectometric signal, measuring equipment resolution, and signal attenuation caused by dielectric losses. Since the measurement uncertainty depends on the length of the measuring rod, the minimum length recommended for TDR moisture measurement is 50 mm [17]. Below this value, the measurement uncertainty increases rapidly due to the applied pulse rise time. The application of shorter probes forces a shorter pulse rise time, which is not convenient due to the decrease of water dielectric permittivity with the frequency [17]. Longer probes increase the measurement accuracy, but the electromagnetic pulse is attenuated along the probe, making them unsuitable for measurements in lossy (saline) materials. For practical reasons, the maximum length of probes used to conduct measurements in wet materials should not be more than 2000 mm [22]. According to Dalton [26], the maximum length of the probe rod designed for loose materials, such as moist soils or salt-containing building materials, depends on the voltage of input and output pulses (*U)*, apparent permittivity (*ε*) of the examined material, and finally, the material apparent conductivity (*σ*). On the other hand, according to Zegelin et al. [27], the minimum length of the probe depends on the rise time of the electromagnetic pulse (t) and apparent permittivity of the material (*ε*) to achieve specific measurement accuracy.

The geometrical parameters of the probe influence the range of its application. The distance between the probe rods affects the impedance of the probe and the volume at which the electric field penetrates the measured material. According to the literature, the distance between probe rods varies from 5 to 100 mm [22]. Small rod spacing results in the short sensitivity range of the probe [17] and a lower impedance of rods. Conversely, large rod spacing increases the sensing volume as well as rod impedance. The amplitude of the incident signal entering the probe is maximal when the electrical impedance of the probe is equal to the cable impedance. The maximum practical spacing of probe rods is about 10 cm [17,27]. An important parameter affecting the electrical impedance of the TDR probe is the ratio between rod spacing *r* and rod diameter *d* (*r*/*d*). According to Knight [17], this ratio should be ten times greater than the measured material grain size in order not to sense the separate grains of the material.

Classical TDR probes have been optimized for measuring moisture in soils in which it is relatively easy to insert measuring rods. In many cases, this is difficult to achieve, and in the case of rigid building materials or rocks, inserting rods is not possible [8,10,28,29,30,31]. In order to insert rods, special holes have to be drilled. This makes the measurement invasive and difficult to apply. Conducting measurements under laboratory conditions requires material sampling for probe installation [30,32].

In order to solve the problems of measuring the moisture content of rigid building materials and constructions, non-invasive surface sensors were applied. A non-invasive TDR sensor made of curved long brass waveguides covered by an acrylic plate was proposed by Selker et al. [33]; it required individual calibration for moisture measurement. The standard error of this sensor was determined to be ±0.02 cm^3^/cm^3^. Perrson and Berndtsson [34] proposed a probe in which standard three-rod TDR probes were covered with a dielectric material with deliberately prepared semi-circular holes. This design enabled the non-invasive determination of the moisture content of the porous material. The surface TDR sensor designs were presented by Wraith et al. [35], where a moisture sensor was proposed to measure the surface soil layer. The sled-shaped sensor was dragged along the soil surface to measure the moisture content of the upper soil layer. Another idea of a TDR surface sensor for measuring moisture in solid materials was proposed in a Polish patent claim [36]. Its prototype and application possibilities have been presented in subsequent papers [18,37] by the authors of this article.

In the literature, there is little information about the sensing volume or sensing range of the TDR surface probes. Many investigations present theoretical or laboratory considerations [17,22,27,38,39] only for invasive TDR probes, considering the electrical probe field that enters the sample [40]. Another possibility is that the information about probe sensitivity volume is provided by the sensor producer [41]. Theoretical analysis of pulse propagation in a system surface probe and the measured material is more difficult due to parallel propagation of the pulse in both the measured material and in the probe [42]. It should be emphasized that the probe sensitivity range is an important technical property that must be considered during laboratory or in situ investigations or calibration procedures and sample preparations. The probe sensitivity range should be also considered as an error source when the electrical permittivity of the sample varies significantly in space.

This paper presents the investigation results of two TDR surface sensor designs according to [36], which were developed for assessing the moisture content of rigid building materials and barriers. The presented designs differ in the spacing of the two measuring elements. The motivation of the study was to evaluate the sensitivity of TDR surface sensors to moist material layer width. The measurement was performed for various moisture contents and widths of measured material for both sensors. This evaluation is based on laboratory experiments on building material samples and statistical evaluation using two-way ANOVA and post hoc methods [43,44].

## 2. Materials and Methods

### 2.1. Description of the Surface TDR Sensors Design

**Figure 1:**
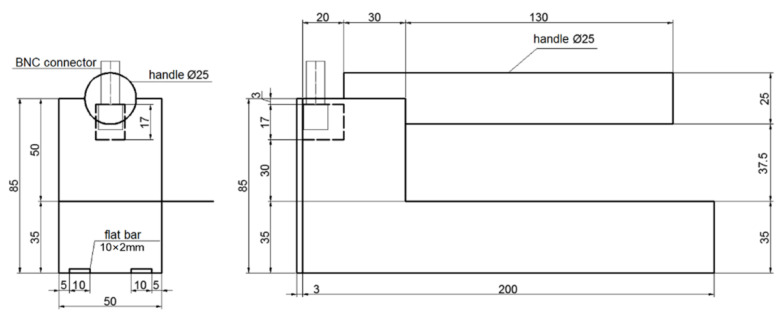
Sensor A design [18].

**Figure 2:**
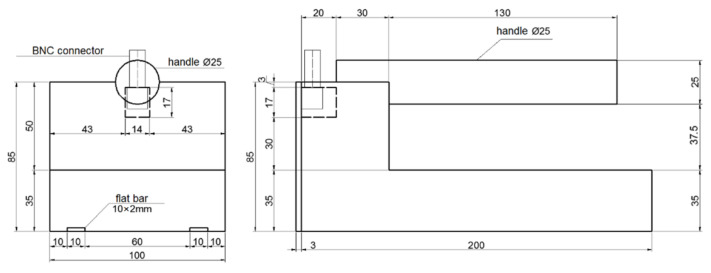
Sensor B design.

**Figure 3:**
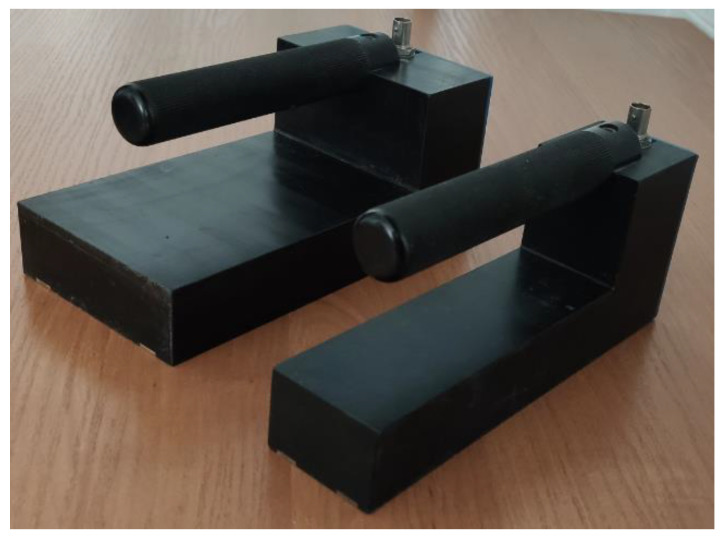
Photographs of both sensors used in the experiment.

For the experiment, two designs of TDR sensors were utilized. Both designs are presented in Figure 1, Figure 2 and Figure 3. They are made of black polyoxymethylene (POM), which is characterized by an apparent permittivity value equal to 3.8 [45]. The measuring elements of both sensors designs were made of 10 × 2 mm brass plate bars. Data transmission between the sensor and the TDR multimeter was provided by a coaxial cable with an impedance of 50 Ω. The coaxial cable was connected with the measuring elements by means of BNC connectors and a simple printed circuit having only two lines, which were soldered to the measuring elements and to the BNC connector pins. The coaxial cable was terminated with an SMA connector on the other end to connect the sensors to the TDR multimeter.

Both sensors differed in spacing between two measuring elements. The first one, previously described in the article [18] (Sensor A), was characterized by the distance between brass bars equal to 30 mm (Figure 1) while the second one (Sensor B) was characterized by the distance between brass bars equal to 70 mm (Figure 3), which was measured between the centers of the bars. The length of both sensors was 200 mm (length of the measuring elements), whereas width, which depended on the brass bars spacing, amounted to 50 mm (Sensor A) and 100 mm (Sensor B).

Figure 1 presents the design of the Sensor A prototype, while Figure 2 presents the design of the Sensor B prototype.

Figure 3 presents the photograph of Sensors A and B.

Both sensors are controlled by a TDR multimeter (ETest, Lublin, Poland). The signal emitted by the multimeter is transmitted by the coaxial cable to the sensors via BNC connectors and then reaches the measuring bars being in contact with the measured material. It propagates along the measuring elements and is reflected from their terminations to return to the TDR multimeter, where the waveform that can be recalculated into the apparent permittivity of the material is collected. The interpretation of waveforms, together with sensor calibration, uncertainty evaluation, and laboratory tests, were described in the authors’ previous article [18].

### 2.2. Description of the Tested Material and Sample Preparation

The tested building material was autoclaved aerated concrete, which is a popular material used for building houses. The major feature of the material is high open porosity and high water absorptivity. The basic parameters of the material are given in Table 1.

**Figure 4:**
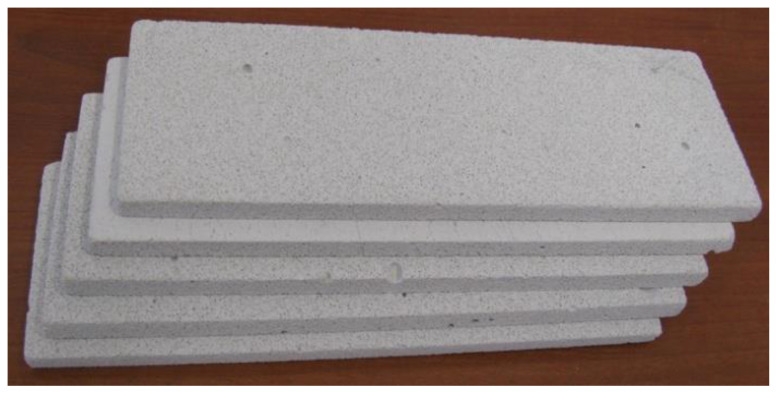
Set of aerated concrete samples used for the experiment.

**Figure 5:**
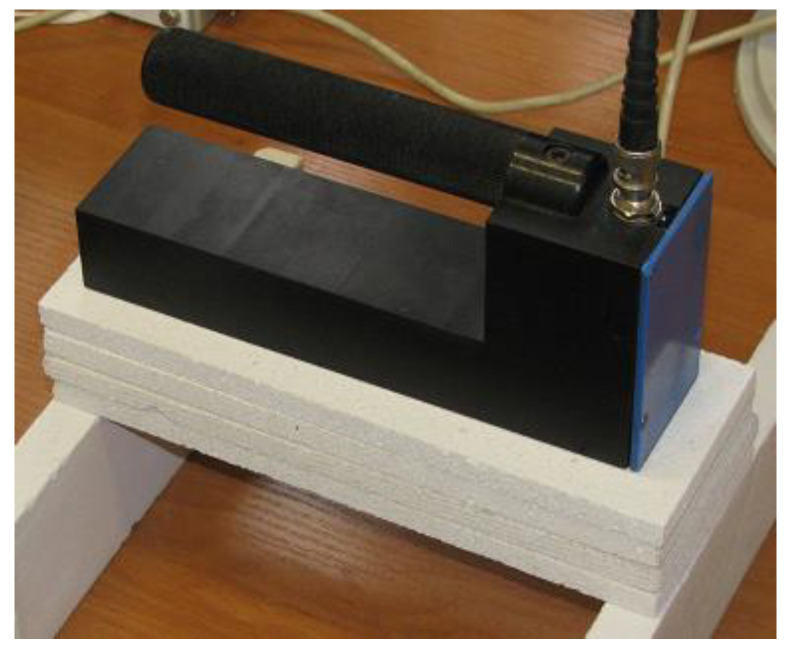
Sensor A during the experiment.

Two 10-element sets of samples were prepared for the investigations. The samples were prepared mechanically by cutting and polishing using fine sandpaper to achieve a flat and smooth surface. The dimensions of the samples used for Sensor A were 230 × 80 × 10 mm and in the case of Sensor B 230 × 130 × 10 mm with the thickness of each sample equal to 10 mm. In order to determine the sensor sensitivity depending on moisture, each sample was dried in an oven to dry mass and then gradually moistened to reach the maximum water content level 0.3 cm^3^/cm^3^ (30.0%_vol_) with 5% intervals. Low sample thickness and water parameters of the material enabled achieving the maximal available homogeneity of the water distribution inside the material sample. Figure 4 presents the samples used for the experiment, and Figure 5 shows Sensor A with material samples during measurement.

### 2.3. Description of the Experimental Setup

For the described research, the following measuring setup was applied:

- TDR multimeter with multiplexer (LOM, ETest, Lublin, Poland);
- Surface TDR sensors—Sensor A and Sensor B (own design);
- PC for TDR meter control and data processing;
- Software to control the TDR meter and post-process the obtained data (own design);
- Two sets of aerated concrete samples;
- Laboratory scales (WPT 6 C/1, Radwag, Poland);
- Drying oven (Memmert VO-500).

For the study, it was assumed that the sensor response would depend on the electrical properties of the material (dry to saturated), the surrounding air (apparent permeability equal to 1), and the dielectric (polyoxymethylene). The limited range of the electromagnetic signal in the tested material would cause no increase of the apparent permittivity value at a certain thickness depending on the material moisture. This allows the interpretation that the sensor sensitivity equals the actual sample thickness.

In order to determine the sensitivity of the sensor, it was necessary to collect the signal response from both TDR sensors and observe the increase in signal propagation time along the measuring elements as both the total sample thickness and the aerated concrete moisture content increased. The readings on each configuration were repeated 5 times, and the data presented in the results section are the average of the measurements.

The first measuring sessions were conducted on empty sensors, which enabled evaluating the apparent permittivity of the ambient air. For the next step, a single 10 mm thick sub-sample was used, having 5%_vol_ (0.05 cm^3^/cm^3^) material moisture, and subsequently, 10 mm thick samples were added to achieve a total sample thickness of 10 cm. In the next stage of the experiment, the samples were moistened to 10%_vol_ (0.1 cm^3^/cm^3^), and the previous steps were repeated. The research continued for the following sample moisture: 15%_vol_, 20%_vol_, 25%_vol_ (0.15 cm^3^/cm^3^, 0.2 cm^3^/cm^3^, 0.25 cm^3^/cm^3^) and was terminated after examining the saturated samples (30%_vol_–0.3 cm^3^/cm^3^). The measurement was conducted under constant conditions at a temperature of (20 °C ± 1 °C) and air relative humidity of 45% ± 5%.

### 2.4. Description of Analysis Methods

The analysis of variance (two-way ANOVA) was used as the main statistical tool. ANOVA is employed to compare the mean of multiple groups; more precisely, two-way ANOVA is applied to simultaneously evaluate the effect of two different grouping variables on a continuous outcome variable.

Two-way ANOVA examines the influence of two independent classifying factors divided into many levels on the values of the analyzed measurable feature. It is an extension of the one-way ANOVA method, which checks whether one independent variable affects the results of one dependent variable.

In the two-way ANOVA method, if there are at least two observations in each subgroup formed by the simultaneous division into levels due to both factors, the influence of the interaction of the two considered features on the variability of the measurable feature is also examined.

The method compares the between-group variance with the within-group variance. It is important that the between-group variance should be large and the within-group variance should be as small as possible. The necessary assumptions of the analysis of variance are the normality and homogeneity of variance in subgroups determined by the levels of classifying factors.

The first description of the two-way ANOVA method appeared in 1925 in Fisher’s Statistical Methods for Research Worker [47]. In 1934, Yates [48] described the procedures for the unsustainable case, which were subsequently corrected many years later by Fujikoshi [43]. In 2005, Gelman [49] proposed a multi-level model in the ANOVA method. The one-way ANOVA and two-way ANOVA methods are commonly used in scientific research, including in the field of medicine, social sciences, and finance [44,50,51].

ANOVA only indicates if the overall influence of the independent variables on the expected outcome is significant along with their relationship to the outcome itself. In order to investigate where the differences appear, post hoc tests should be used, which is why in this research, the ANOVA test was supplemented with the most popular and widely used Tukey’s HSD (Honest Significant Difference) post hoc test to identify homogenous groups [52]. The aim of partition into homogenous groups is to identify the groups where each pair has significantly different means on the dependent variable. It can be interpreted as elements contained in the same group behave similarly, while those lying in different groups do not.

A partial eta squared ($\eta_{p}^{2})$ was used as the measurement of the effect size. It expresses the sum of squares of the effect in relation to the sum of squares of the effect and the sum of squares of the error associated with the effect. The partial eta squared is calculated as follows:

(1) $$\eta_{p}^{2}=\frac{SS_{effect}}{SS_{effect}+SS_{error}}.$$

Following Field [53], if $\eta_{p}^{2}<0.01$, the effect size is considered as very small, if $0.01\leq \eta_{p}^{2}<0.06$, the effect size is considered as small, if $0.06\leq \eta_{p}^{2}<0.14$, the effect size is considered as medium, and finally, if $\eta_{p}^{2}\geq 0.14$, the effect size is considered as large.

All statistical analyses and visualizations of results were performed in the R statistical environment [54] along with a number of packages extending the capabilities of the basic program [55,56,57,58].

## 3. Results

**Figure 6:**
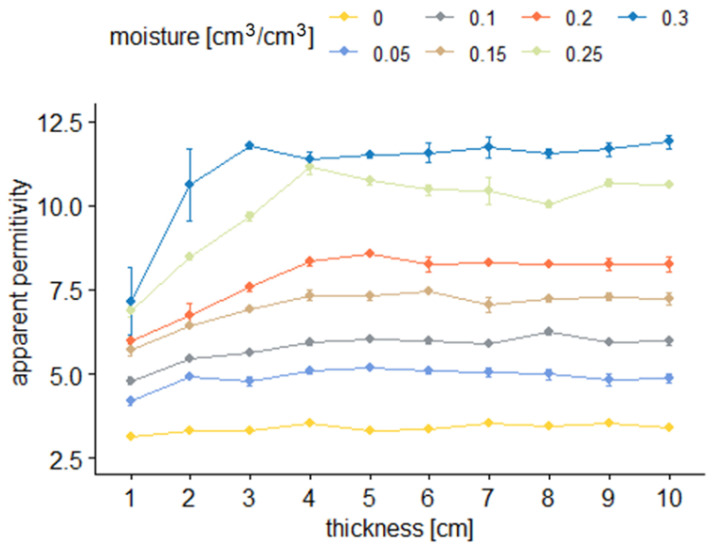
Average apparent permittivity readouts with sensor A depending on sample thickness for the particular material moisture.

**Figure 7:**
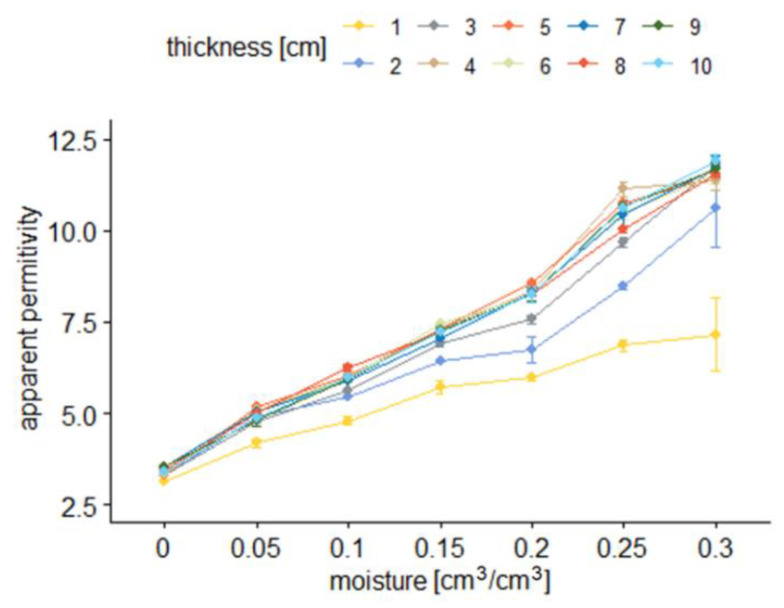
Average apparent permittivity readouts with sensor A depending on material moisture for particular sample thickness.

Figure 6 and Figure 7 present the averages of the TDR readouts of apparent permittivity (*ε*) depending on material volumetric water content (*θ*) and sample thickness for sensor A.

**Figure 8:**
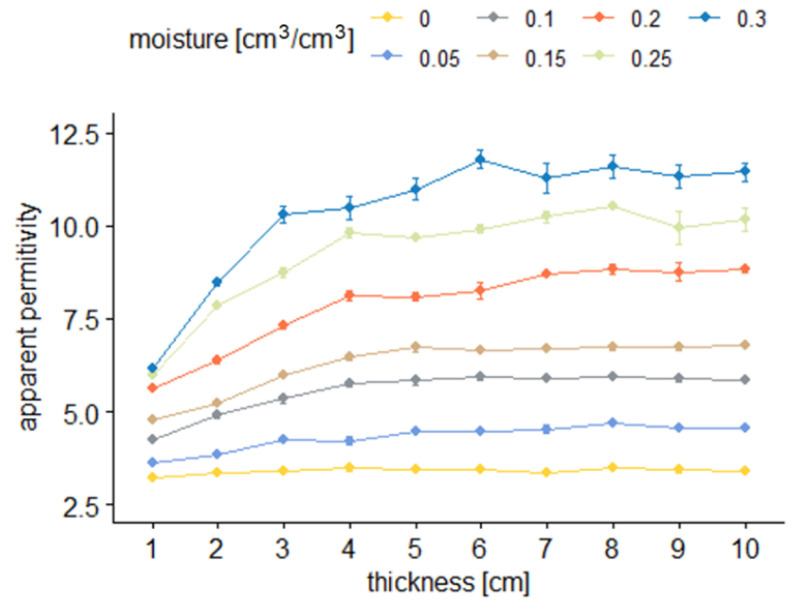
Average apparent permittivity readouts with sensor B depending on sample thickness for particular material moisture.

**Figure 9:**
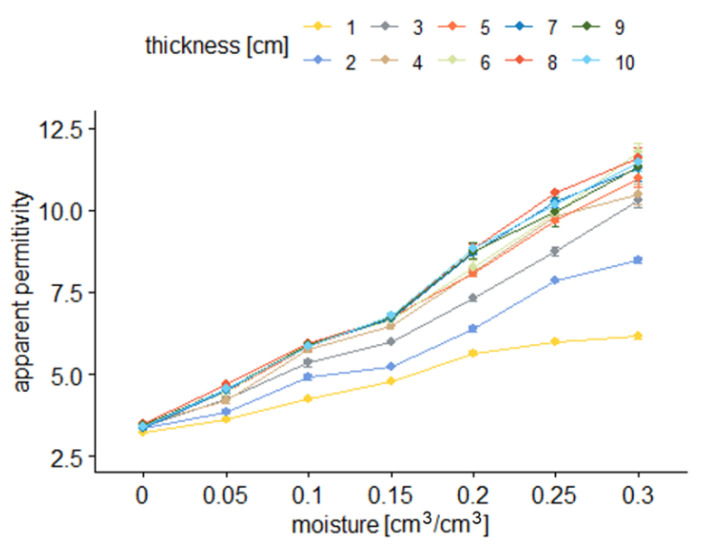
Average apparent permittivity readouts with sensor B depending on the material moisture for particular sample thickness.

Figure 8 and Figure 9 present the TDR readouts of apparent permittivity (*ε*) depending on the material moisture (*θ*) and sample thickness for sensor B.

Figure 6 and Figure 8 illustrate the dependence between the average values of apparent permittivity read by two tested sensors and sample thickness at different levels of sample moisture. In the figures, dots denote averages of respective groups. They are presented with a range of standard error. Colors correspond to different moisture levels.

Figure 7 and Figure 9 show how the averages of apparent permittivity are dependent on sample moisture for different sample thicknesses. In the figures, dots denote averages of respective groups. They are presented with a range of standard error. Colors correspond to different sample thicknesses.

## 4. Discussion

From the results presented in Figure 6, Figure 7, Figure 8 and Figure 9, it is visible that the effective apparent permittivity value increases together with sample thickness. The increase is more visible for the samples with higher moisture states. In the case of dry samples, the apparent permittivity changes read by the TDR instrument is not visible. A rapid apparent permittivity increase in moist samples can be explained by the presence of water within the sensor sensitivity range, and the water present out of reach of the sensor did not influence the permittivity readouts, which was observed in Figure 6 and Figure 8 as constant values. In the case of dry samples, this phenomenon was not so visible due to the small differences between the sample material permittivity and ambient air.

In the case of the air-dried samples, both sensors indicate a constant value of apparent permittivity approximately equal to 3.0, which is independent of sensor design and sample thickness. On the other hand, the water present in the tested material changes its electric properties, and the increase of apparent permittivity becomes noticeable. In the case of the 0.05 cm^3^/cm^3^, the 1 cm thick aerated concrete sample shows the increase of the apparent permittivity value to reach about 4.0 in the case of sensor A and 3.6 in the case of sensor B. Progressive sample thickness increase caused the small increase in the apparent permittivity value to reach 5.0 in the case of sensor A and 4.5 in the case of sensor B. Higher values of material moisture increased the values of the apparent permittivity read by the TDR instrumentation to reach 6 for a fully saturated 1 cm thick sample, in the case of both sensors and more than 11.0 for the samples thicker from 3 to 4 cm. In the case of both sensors, it was noticed that for moist samples, the apparent permittivity increase was rapid for small sample thicknesses, and after reaching a critical point, its value was maintained at a constant level. These dependences were developed into mathematical formulas presented in Table 2, Table 3, Table 4 and Table 5 and are discussed widely in the next paragraphs.

Table 2 and Table 4 present the dependences between sample thickness and apparent permittivity values read by the TDR instrumentation. In the case of dry material, linear or nearly constant models were assigned, even though small values of coefficient of determination were obtained. Any moisture appearance increased the curvature of the dependences but also raised the values of *ε*, which is visible by increasing the values of regression coefficients. More valuable information could be read from Table 3 and Table 5 where linear regression models were assumed to describe all the dependences between material moisture and apparent permittivity read by the TDR meter. In the case of both sensors, the coefficient of determination values are high: 0.835–0.990 for sensor A and 0.971–0.990 for sensor B. Interestingly, the regression slopes are rising from about 13 to 28 for sensor A, maintaining the constant value between 26 and 27 when the sample thickness of 3 cm was achieved. The y-intercept values for all readouts are nearly constant and approximate 3.3. On the other hand, for Sensor B, the y-intercept values are lower, ranging between 3.09 and 3.24, and the regression slopes rise from 10.6 to 27.8, but interestingly, this increase is noticeable for the thicknesses of 1–5 cm; for the thicknesses of 6–10 cm, the regression slopes are nearly constant.

For precise evaluation of the range of the sensor, the data discussed were interpreted through the analysis of variance (two-way ANOVA with the moisture (cm^3^/cm^3^) and thickness of the sample (cm) as the main factors as well as their interaction), which is used to determine if the differences between the average values of the apparent permittivity are not accidental. The number of repetitions within the sub-group equaled 3. No outliers were identified. Variance homogeneity was confirmed by Levene’s test. Although the Shapiro–Wilk test rejected the assumption of the normality of distribution of residuals following Lindman [59], who showed that F-statistics is fully robust to the failure of this assumption, the results of the ANOVA test can be considered as reliable. Zero hypothesis assumes the values of the group averages p of the population are the same: equal to the general average value. Actually, three zero hypotheses were tested:

- Equality of the group averages for the main factor—moisture;
- Equality of the group averages for the main factor—thickness;
- Equality of the group averages for the interaction of moisture and thickness.

The ANOVA test showed the significance of the differences between the average values of the homogenous groups in all three cases at the level of *p* < 0.001.

As all zero hypotheses were rejected for both narrow (A) and wide (B) TDR sensors, Tukey’s HSD post hoc test was conducted to show the statistically significant differences between the particular sub-groups (samples moisture and thickness) to find out which means of specific groups (compared with each other) are different. The test compares all possible pairs of means. It can be stated that the samples constitute a homogenous group if the differences in the averages of apparent permittivity are not statistically significant.

In the case of sensor A, the ANOVA suggests the following:

- The main effect of moisture is statistically significant and large (F(6, 140) = 1498.23, *p* < 0.001; $\eta_{p}^{2}$ = 0.98, 90% CI (0.98, 0.99));
- The main effect of thickness is statistically significant and large (F(9, 140) = 63.77, *p* < 0.001; $\eta_{p}^{2}$ = 0.80, 90% CI (0.76, 0.84));
- The interaction between moisture and thickness is statistically significant and large (F(54, 140) = 6.05, *p* < 0.001; $\eta_{p}^{2}$ = 0.70, 90% CI (0.58, 0.72)).

The fact that in each case, $\eta_{p}^{2}$ is much greater than 0.14—which means that the effect size is very large—should be emphasized. In addition, this fact is confirmed by narrow confidence intervals.

**Figure 10:**
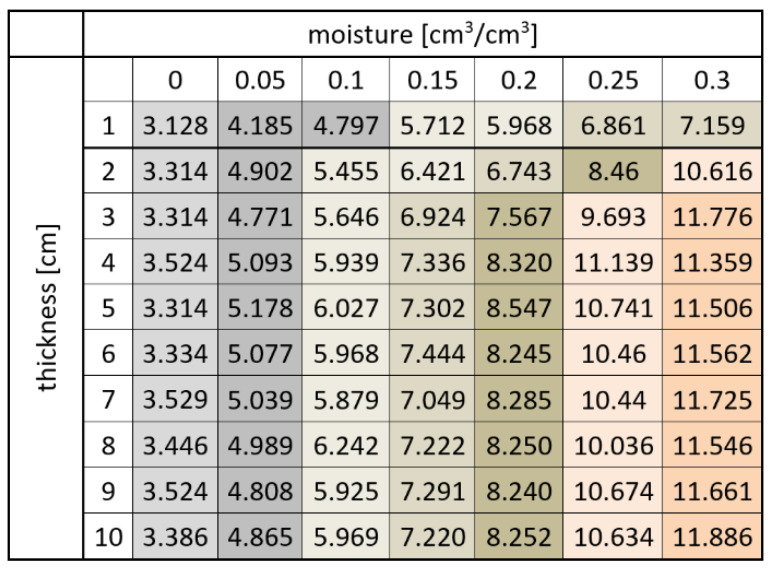
Post hoc analysis of apparent permittivity readouts using sensor A for various values of sample moistures and sample thicknesses. Each color means different homogenous group.

Figure 10 shows the post hoc test results for the TDR sensor A. Seven homogenous groups are distinguished based on the similarities and dissimilarities between averages of apparent permittivity for different combinations of sample moisture and thickness. In the figure, different colors were used to mark the division into homogeneous clusters, which result from the influence on the apparent permittivity of both factors (sample moisture and thickness) and their interaction.

In the case of sensor B, the ANOVA suggests the following:

- The main effect of moisture is statistically significant and large (F(6, 140) = 3196.57, *p* < 0.001; $\eta_{p}^{2}$ = 0.99, 90% CI (0.99, 0.99));
- The main effect of thickness is statistically significant and large (F(9, 140) = 241.16, *p* < 0.001; $\eta_{p}^{2}$ = 0.94, 90% CI (0.93, 0.95));
- The interaction between moisture and thickness is statistically significant and large (F(54, 140) = 18.71, *p* < 0.001; $\eta_{p\;}^{2}$ = 0.88, 90% CI (0.84, 0.89)).

Similarly, as for sensor A, the fact that in each case, $\eta_{p}^{2}\;$ is much greater than 0.14—which means that the effect is very large—should be noted. Moreover, this fact is confirmed by narrow confidence intervals. These effects are even larger than in the case of sensor A.

**Figure 11:**
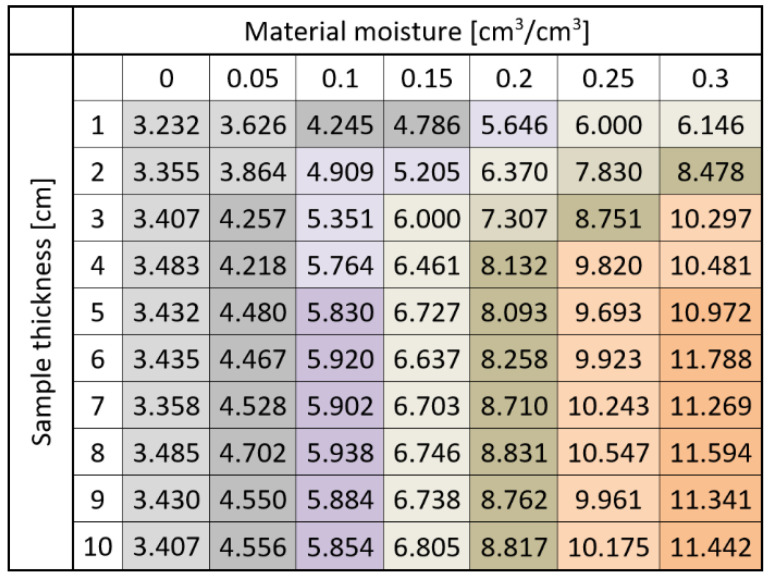
Post hoc analysis of apparent permittivity readouts using sensor B for various values of sample moistures and sample thicknesses. Each color means different homogenous group.

Figure 11 shows the post hoc test results for the TDR sensor B. Nine homogenous groups were distinguished based on the similarities and dissimilarities between averages of apparent permittivity for different combinations of sample moisture and thickness.

Using the ANOVA and Tukey post hoc tests, it was possible to evaluate the maximal range of sensitivity of both sensors. After reaching the exact value depending on material moisture and sample thickness, the increase in apparent permittivity was not measured. From the results presented in Figure 10 representing sensor A, it was noticed that in the case of the dry and low moisture containing samples (0 and 0.05 cm^3^/cm^3^), all permittivity readouts belong to the same homogenous groups (another one for air dry, another one for 0.05 cm^3^/cm^3^ moist). This means that in the case of dry samples, it is not possible to evaluate the range of sensor influence using the method proposed. An increase of material moisture causes more inhomogeneous apparent permittivity distribution, and more homogenous groups could be distinguished. Two groups are observed for moistures of 0.1 and 0.15 cm^3^/cm^3^. Interestingly, for 0.1 cm^3^/cm^3^ moist material, one group is distinguished for the 1 cm thick sample, and the rest of the samples are in the same group; in the case of 0.15 cm^3^/cm^3^, the first group consists of 1 and 2 cm apparent permittivity readouts, and the second ones are in the second homogenous group. For more moist samples, the values of apparent permittivity are higher, which results in the greater number of the homogenous groups; nevertheless, it could be noticed that two first sample thicknesses (1 and 2 cm) are in separate groups, whereas between 3 and 10 cm, they are in homogenous groups, which are different from each other. When looking at the rows representing sample thickness, it should be noticed that after 3 cm, the homogenous groups are distributed similarly with the same pattern. For less thick samples, division for homogenous groups is not so clear (e.g., for thickness of 1 cm, the same homogenous groups are assigned despite different moisture values: 0.05 and 0.1 (cm^3^/cm^3^) as well as 0.25 and 0.3 (cm^3^/cm^3^)). These observations allow assuming that the maximum sensitivity range of the investigated sensor starts from 3 cm.

In the case of sensor B, the situation is similar, but the differences between the homogenous groups are more visible. In the case of the dry samples, only one homogenous group was distinguished independently of the sample size. In the 0.05 cm^3^/cm^3^ moist samples, two groups can be distinguished, i.e., 1–2 cm and 3–10 cm thick. For the 0.1 cm^3^/cm^3^ material, three groups could be distinguished, with the largest between 5 and 10 cm. A similar situation but for higher values of apparent permittivity values is noticed for saturated samples (0.3 cm^3^/cm^3^). In the case of material moistures equaling 0.2 and 0.25 cm^3^/cm^3^, the largest homogenous groups are between 4 and 10 cm. Considering the rows, one can see similar dependencies as in the case of Figure 10, but the constant pattern of homogenous groups distribution starts from 5 cm. For lower sample thickness, this division is not so clear. These observations allow assuming that the maximum sensitivity range of the investigated sensor starts between 4 and 5 cm and is deeper compared to sensor A.

Both the regression formulas presented in Table 2, Table 3, Table 4 and Table 5 and two-way ANOVA analysis confirmed similar dependences between material moisture, sensor rods spacing and measuring sensitivity.

The data achieved within the investigation are important for the process of sensor development. First of all, for the calibration procedure, the minimal thickness of the sample must be larger compared to 3 cm for sensor A and 5 cm for sensor B. Smaller thickness of the sample may lead to underestimation of the apparent permittivity value by the TDR instrumentation. Greater thickness hinders achieving a proper homogeneity of moisture distribution in the sample. Secondly, both laboratory and in situ investigations should consider that the apparent permittivity and thus moisture values are only true for the subsurface zone of the material (3 cm in the case of sensor A and 5 cm in the case of sensor B).

The differences of the sensor influence estimated within the conducted research for both designs (A and B) confirm the literature reports concerning the invasive TDR probes [17,60] that the capacity of their influence increases together with measuring rods distribution. Comparing the achieved values to other available invasive TDR probes, it can be assumed that they are similar to those declared by some of the producers. For example, commercial FP/mts probes by ETest, Lublin, and Poland probes, having the transmission lines separated by 16 mm, have the region of influence defined as a cylinder with an approximate diameter of 5 cm circumference around the sensor rods [41]. On the other hand, according to the same source, laboratory LP/ms sensors with transmission lines separated by 5 mm are characterized by the region of influence equal to 5 mm.

Comparing the achieved results to other non-invasive sensors presented by the co-authors of this article, it should be emphasized that they are similar to the values presented in [18] for the TDR sensor with a similar construction (between 30 and 40 mm) and nearly the same as achieved by the simple TDR sensor presented in the following research [9]: 40 mm of signal range for the average sensors distribution equal to 50 mm.

## 5. Conclusions

The research on the signal range of two designs of the non-invasive TDR sensors that differed in measuring rods distribution under various levels of moisture of the tested material enabled formulating the following conclusions:

- The range of both sensors influence does not exceed 50 mm deep into the examined material.
- The space between measuring rods of the TDR sensor influences the range of sensor influence. In the case of the narrow sensor A, with the span between two rods equal 30 mm, its range is approximately 30 mm. In the case of the wider design with a rod spacing of 70 mm, its range of effect reaches 50 mm.
- Material moisture influences the range of the sensitivity of the tested sensors.
- Estimation of signal influence in the case of dry or low-moisture material was not possible; only one homogenous group was distinguished in post hoc tests.
- In the case of the medium-saturated material, it was possible to distinguish two homogenous groups, and the sensor sensitivity reached between 10 and 20 mm.
- In the case of highly saturated samples, the sensor sensitivity range was the deepest.
- The achieved values should be treated as technical data and used for proper calibration procedures as well as proper conduct of laboratory and in situ measurements.

## Figures and Tables

**Table 1:** Basic moisture parameters of aerated autoclaved aerated concrete applied in the experiment were determined according to standard [46].

Parameter	Unit	Value
Bulk density	(kg/m^3^)	620
Maximum volumetric water absorptivity	(cm^3^/cm^3^)	0.3

**Table 2:** Mathematical dependences between sample thickness (cm) and apparent permittivity values read by Sensor A, for particle sample moisture.

Moisture (cm^3^/cm^3^)	Regression Model	R^2^
0	*ε* = 0.0272 *d* + 3.232	0.403
0.05	*ε* = −0.0282 *d*^2^ + 0.3488 *d* + 4.0597	0.548
0.1	*ε* = −0.0302 *d*^2^ + 0.4326 *d* + 4.5677	0.821
0.15	*ε* = −0.0427 *d*^2^ + 0.5933 *d* + 5.3722	0.756
0.2	*ε* = −0.0663 *d*^2^ + 0.9359 *d* + 5.2477	0.841
0.25	*ε* = −0.0986 *d*^2^ + 1.3801 *d* + 6.1187	0.774
0.3	*ε* = −0.0985 *d*^2^ + 1.3863 *d* + 7.2492	0.545

*ε*—apparent permittivity; *d*—sample thickness (cm).

**Table 3:** Mathematical dependences between sample moisture (cm^3^/cm^3^) and apparent permittivity values read by Sensor A for particle sample thickness.

Thickness (cm)	Regression Model	R^2^
1	*ε* = 13.299 *θ* + 3.4066	0.835
2	*ε* = 21.648 *θ* + 3.3116	0.882
3	*ε* = 26.535 *θ* + 3.1183	0.970
4	*ε* = 27.127 *θ* + 3.461	0.969
5	*ε* = 27.299 *θ* + 3.4216	0.987
6	*ε* = 26.945 *θ* + 3.3997	0.983
7	*ε* = 26.997 *θ* + 3.3712	0.974
8	*ε* = 26.001 *θ* + 3.4897	0.990
9	*ε* = 27.47 *θ* + 3.3256	0.983
10	*ε* = 28.087 *θ* + 3.2459	0.982

*ε*—apparent permittivity; *θ*—material moisture (cm^3^/cm^3^).

**Table 4:** Mathematical dependences between sample thickness (cm) and apparent permittivity values read by Sensor B for particle sample moisture.

Moisture (cm^3^/cm^3^)	Regression Model	R^2^
0	*ε* = 0.0128 *d* + 3.3317	0.143
0.05	*ε* = −0.0192 *d*^2^ + 0.3097 *d* + 3.3596	0.894
0.1	*ε* = −0.0421 *d*^2^ + 0.6125 *d* + 3.8097	0.925
0.15	*ε* = −0.0464 *d*^2^ + 0.7125 *d* + 4.1503	0.938
0.2	*ε* = −0.0569 *d*^2^ + 0.9584 *d* + 4.8132	0.943
0.25	*ε* = −0.0959 *d*^2^ + 1.4364 *d* + 5.0859	0.907
0.3	*ε* = −0.1251 *d*^2^ + 1.8447 *d* + 5.0501	0.891

*ε*—apparent permittivity; *d*—sample thickness (cm).

**Table 5:** Mathematical dependences between sample moisture (cm^3^/cm^3^) and apparent permittivity values read by Sensor B for particle sample thickness.

Thickness (cm)	Regression Model	R^2^
1	*ε* = 10.636 *θ* + 3.2163	0.971
2	*ε* = 17.687 *θ* + 3.0629	0.973
3	*ε* = 22.58 *θ* + 3.0944	0.978
4	*ε* = 24.69 *θ* + 3.205	0.980
5	*ε* = 25.222 *θ* + 3.2491	0.990
6	*ε* = 27.365 *θ* + 3.0992	0.981
7	*ε* = 27.121 *θ* + 3.1764	0.984
8	*ε* = 27.794 *θ* + 3.237	0.984
9	*ε* = 26.738 *θ* + 3.2272	0.979
10	*ε* = 27.36 *θ* + 3.1896	0.987

*ε*—apparent permittivity; *θ*—material moisture (cm^3^/cm^3^).

## Data Availability

Not applicable.
